# Factors affecting perception of the normal pediatric appendix on sonography

**DOI:** 10.1186/s13089-019-0148-1

**Published:** 2019-12-21

**Authors:** Denise Castro, Joseph Yang, Prasan Patel, Eric Sauerbrei, Wilma Hopman, Mila Kolar, Don Soboleski

**Affiliations:** 1 Department of Radiology, Kingston Health Sciences Centre, Queen’s University, 76 Stuart Street, Kingston, ON K7L 2V7 Canada; 2 Queen’s School of Medicine, Queen’s University, 80 Barrie Street, Kingston, ON K7L 3N6 Canada; 3 WJ Henderson Centre for Patient Oriented Research, Queen’s University, 76 Stuart Street, Kingston, ON K7L 2V7 Canada; 4 Department of Surgery, Kingston Health Sciences Centre, Queen’s University, 76 Stuart Street, Kingston, ON K7L 2V7 Canada

**Keywords:** Appendicitis, Sonographer experience, Pediatric, Perception skills, Radiology residents

## Abstract

**Background:**

To determine if an inherent perception skill along with sonographer experience, knowledge base, scanning time play a role in the identification of the normal appendix in the pediatric population. This is a retrospective review of pediatric (< 18 years old) patients with a clinical suspicion of acute appendicitis presenting to the emergency department of two affiliated academic tertiary care hospitals over a 1-year time span. All patients had a sonogram performed by 1/15 sonographers or by 1/8 on-call radiology residents. Those with a normal or non-visualized appendix with subsequent discharge from ER were included in the study. Patient demographics, minutes spent scanning, and sonographer years of experience in general abdominal scanning and residents level of training were recorded.

**Results:**

Of the 127 patients included in the study, 51 (40%) were male and 76 (60%) were female, with a mean age of 11.8 ± 4.2 years. Sonographers who failed to see a normal appendix had less experience (median 8 years) than those who did visualize the appendix (median 15 years), *p* ≤ 0.001. Longer time spent scanning was also associated with visualizing a normal appendix (20.4 versus 29.1 min, *p* = 0.001). In multivariable logistic regression, more time spent scanning (OR 1.04, 95% CI 1.01, 1.07, *p* = 0.012) and increased sonographer experience (OR 1.07, 95% CI 1.02, 1.13, *p* = 0.012) resulted in greater odds of perceiving the appendix. The top 4 were significantly more likely to visualize the appendix (88.0%) than all of the other combined (20.8%, *p* < 0.001), and they also had substantially more experience (median 15 years versus 8 years, *p* < 0.001). Overall, sonographers were more likely to see a normal appendix (61%) than the residents (14%), *p* < 0.001.

**Conclusion:**

Sonography to rule out appendicitis in the pediatric patient is in general most successful when performed by experienced sonographers with adequate time to perform the scan. Triaging patients to those sonographers who have displayed optimal perceptual ability of the normal appendix may help optimize patient care and hospital resources. Having experienced sonographers available after hours would allow for optimal care in the setting of ‘query’ appendicitis.

## Background

Recognition of diagnostic error and more specifically perception error remains a significant challenge in radiologic practice with the overall prevalence appearing unchanged from the first estimates by Garland in 1949 [1–4]. The process of image interpretation is based on individual complex psychophysiological and cognitive activities not readily observable and subject to a wide variety of error types [5–7]. Studies investigating cognitive and systemic factors contributing to diagnostic error have mostly been focused on assessment of missed breast or lung nodules. Perception in sonography is fundamentally different than the other modalities where images are produced in a formatted nature and then later evaluated by the observer (passive perception). Ultrasound is performed in a less standardized way, requiring the most appropriate acoustic window to evaluate the region of concern. Image production requires the operator’s perceptual skills while acquiring the images (active perception). The objective of this study was to determine if an inherent perception skill along with sonographer experience, knowledge base or scan time was associated with an imager’s ability to perceive the normal pediatric appendix.

## Materials and methods

### Study population

This was a retrospective review of all pediatric patients (< 18 years) sent to the imaging department from the emergency room (ER) of two affiliated academic tertiary care hospitals over a 1-year time span to rule out appendicitis. This centre is a general tertiary care hospital with an embedded pediatric department. A further 1-year review limited to the cases performed by residents was undertaken to acquire more numbers. The sonographic study was performed by one of 15 general sonographers within the diagnostic imaging department or by one of 8 on-call radiology residents. The patients with positive findings for appendicitis or any other identifiable pathology with subsequent treatment were excluded from the database. Patients with a sonogram depicting a normal appendix with subsequent discharge from ER and normal follow-up were included in the study, as were those with an appendix not visualized on sonography with subsequent normal follow-up imaging (CT) and/or discharge from hospital with resolution of symptoms on follow-up. Cases in which there was a discrepancy in the sonographers or residents impression with the attending radiologist were also excluded.

### Ultrasound equipment utilized

Scans were performed on Logic 9 Scanner (GE Medical Systems, Milwaukee, WI), employing a linear 6–15 MHz or convex 1–6 MHz transducer probe. The exams were targeted to the right lower quadrant and performed in a manner consistent with the typical techniques used including graded compression. The initials of the sonographer were used to identify the scanner and data findings extracted from the technical or resident note. The time taken to perform the scan was determined by the time noted on the first obtained image and the last obtained image.

### Statistical analysis

A priori sample size calculations were not done, as this was a convenience sample of all eligible pediatric patients referred to imaging over a 1-year period. Data were collected in an Excel file and imported into IBM SPSS (version 25.0 for Windows, Armonk, New York, 2018) for statistical analysis. Data were initially analyzed descriptively, including frequencies and percentages for categorical data and means and standard deviations for continuous data. The Shapiro–Wilk test was used to assess the normality of the underlying distribution of continuous data. Comparisons of the groups with a normal or non-visualized appendix were made using Chi-square tests for categorical data and independent samples *t*-tests or Mann–Whitney U for continuous data. A multivariable logistic regression model was developed to assess the relative contributions of the variables that had a moderate association (*p* < 0.15) in univariate analysis, while also controlling for patient sex.

## Results

The study group included a total of 127 pediatric patients who were sent to the imaging department over a 1-year time span with a clinical suspicion of acute appendicitis. Of these, 60 (47%) had a normal appendix (6 mm caliber or less with no adjacent inflammatory changes) visualized on sonography and 67 (53%) patients underwent sonography with the appendix not visualized. The population consisted of 76 female and 51 male patients. Table 1 outlines the association between scanner experience, amount of time spent scanning and resident level of training with the ability to perceive the normal appendix on sonography.

**Table 1:** Description of sample and univariate results

Characteristics	Normal appendix *N* = 60	Appendix not visualized *N* = 67	*p* value
Patient sex			
Male *n* (%)	27 (45.0)	24 (35.8)	0.292
Female *n* (%)	33 (55.0)	43 (64.2)	0.113
Patient age (mean ± SD)	12.5 ± 3.5	11.2 ± 4.7	0.001
US time in minutes (mean ± SD)	29.1 ± 15.5	20.4 ± 12.6	< 0.001
Experience in years (median, 25th and 75 percentile)	15 (9, 15)	8 (3, 14)	
Expertise			
Technician *n* (%)	54 (91.5)	35 (53.8)	< 0.001
Junior residents ≤ 3 *n* (%)	3 (5.1)	20 (30.8)	
Senior residents 4+ *n* (%)	2 (3.4)	10 (15.4)	

Numbers do not always add up to overall totals due to small amounts of missing data

*p* values are based on the Chi-square test (sex), independent samples *t*-test (patient age as continuous, US time in minutes) and the Mann–Whitney U (experience in years)

The sonographers who were unable to perceive the normal appendix had significantly less experience (median 8 years) than those who did visualize the normal appendix (median 15 years), *p* < 0.001. The minutes spent performing the scan was significantly shorter for those where the appendix was not visualized (mean 20.4 ± 12.6 min) as compared to the time spent when able to perceive a normal appendix (mean 29.1 ± 15.5), *p* = 0.001. These two variables were significantly and positively correlated; those with more scanning experience took more time to do the assessment (*r* = 0.310, *p* = 0.001).

Multivariable logistic regression (Table 2) demonstrated that increased minutes spent scanning was significantly associated with greater odds of perceiving the normal appendix (OR 1.04, 95% CI 1.01, 1.07, *p* = 0.012). Increased sonographer experience was also significantly associated with greater odds of seeing the appendix (OR 1.07, 95% CI 1.02, 1.13, *p* = 0.012). A patient of older age and male sex was also associated with greater odds, but failed to reach statistical significance (*p* = 0.43 and *p* = 0.099, respectively).

**Table 2:** Multivariable logistic regression model (0 = not seen, 1 = visualized)

Model for pediatrics (age < 18)	Odds ratio (95% confidence interval)	*p* value
Age in years	1.04 (0.94, 1.15)	0.430
Patient sex (0 = male (ref), 1 = female)	0.50 (0.22, 1.14)	0.099
US time in minutes	1.04 (1.01, 1.07)	0.012
Experience in years	1.07 (1.02, 1.13)	0.012

Cox and Snell R-square = 0.17; overall model Chi-square = 22.0, *p* < 0.001

When comparing junior (PGY2/3) to senior radiology residents (PGY4/5), the junior residents were somewhat less successful in identifying the normal appendix (3/23 or 13.0% as compared to 2/10 or 16.7%); however, this was not statistically significant (*p* = 0.771) in this small subset of 35 observations.

Independent of experience, there was a marked difference among the 15 sonographers in their ability to perceive the normal appendix. However, the large number of low-frequency cells in the descriptive table made the tests of significance invalid. Table 3 illustrates a comparison among the 4 sonographers with the highest success in perceiving the normal appendix, to the remainder of the sonographers and the residents in the study. The top 4 were significantly more likely to visualize the appendix (88.0%) than all of the other combined (20.8%, *p* < 0.001), and also had considerably more years of experience (median 15 versus 8 years). Sonographers in general were more likely to see a normal appendix (60.7%) than the residents (14.0%, *p* = 0.001).

**Table 3:** Comparison of top 4 sonographers to all others

	Top 4 sonographers, *N* = 50	All others, *N* = 77	*p* value
US result appendix normal, *n* (%)	44 (88.0)	16 (20.8)	< 0.001
Appendix not visualized, *n* (%)	6 (12.0)	61 (79.2)	
Years of experience, median (25th, 75th percentile)	15 (9, 15)	8 (3, 14)	< 0.001

The *p* value is based on the Pearson Chi-square test and the Mann–Whitney U

## Discussion

Diagnostic/perception error has been defined as a diagnosis that is missed, delayed or wrong as determined by a subsequent definitive test or finding. The lack of perception of a normal finding may also constitute a diagnostic error when clinically relevant. Initial work by Garland in 1949, who estimated an average radiology perception error rate of up to 30%, has led to extensive study in human perception and factors in perception error which, for the most part, remain poorly understood [4, 7, 8]. Increasing workloads, cognitive biases, systemic factors such as lighting, along with rising quality expectations are potential yet minor factors that contribute to perception error, often the result of mental or visual fatigue [1, 3, 5]. Attempts to improve error by work hour alterations, improved environmental setting such as decreased interruptions and changes in luminescence of monitors have had limited success [5].

Errors in imaging have been classified into two broad categories comprising errors in perception versus interpretive errors. Perception error accounts for 60–80% of radiologists errors in clinical practice. The majority lacks any identifiable cause [5, 9]. The persistence of investigative studies depicting the degree of perceptual error among imagers worldwide despite the level of training or experience and spanning multiple modalities would suggest that errors related to carelessness, negligence or underperformance of some kind are not the typical cause. Rather, there appears to be an inherent misperception or under perception associated with the complex process of image interpretation perhaps below the threshold of conscious awareness [5]. More recent studies have further defined perceptual error into three basic types. These include (1) scanning error—a failure to fixate on the adequate region; (2) recognition error—a fixation on the region but for inadequate time and (3) decision-making error—fixation on the appropriate region for adequate time, but an unexplained lack of recognition of pathology [3]. We believe that a lack of perception of a normal finding during the scanning process, rather than the usual lack of recognition on a set of provided images, that will alter patient care and management should be included in this third type.

Previous studies have shown the value of sonographer experience in patient diagnosis [10–12]. Our study hopes to also illustrate the role of an inherent perceptual skill that some sonographers display that can affect patient care. Missed and misinterpreted diagnosis in sonography are often too easily attributed to technical factors such as body habitus or overlying bowel gas. While these factors may limit an examination, the sonographer’s ability to adequately perceive anatomic fascial planes in the region of concern will often determine the ultimate clinical value of the study. Intuitively, one would expect that increased experience (years of training) in scanning or increase in knowledge base (as one would expect the senior residents to have gained more imaging knowledge than the juniors) would correlate with an improved ability to perceive the normal appendix. We found this to be the case, as our junior residents demonstrated a lower ability to identify the normal appendix as compared to their more senior colleagues. Although in general, sonographers with more years of experience correlated with an increase in perceiving the normal appendix, within the group of experienced sonographers there was a wide discrepancy in frequency of perception of a normal appendix suggesting there may be an inherent individual perceptual skill. Just as some radiologists are able to more quickly spot the lung nodule on chest radiographs or the individual who is able to perceive “Waldo” more easily than others, we suggest that there are sonographers that find the normal appendix more easily than others [13].

Utilizing the “query” appendicitis ER population illustrates the potential clinical effect of this difference in sonographer perceptual skill. Demonstration of a normal appendix on sonography can, for the most part, eliminate this potentially dangerous disease process from the physician’s list of concern, potentially saving the patient from further studies such as CT/MR and expediting patient care through the emergency department. Interestingly, if all the patients sent to the imaging department to rule out appendicitis could have been triaged to the four sonographers with the greatest success in perceiving the normal appendix, an additional 50 patients would have had their appendix visualized, and the overall percentage of cases where the appendix was not seen would drop from 52 to 12%. As such, it may be of benefit for less experienced sonographers to accompany sonographers who are more experienced to gain experience and technical skills in visualizing the appendix. Alternatively, this discrepancy in sonographer perceptual skill may suggest that triaging query appendicitis cases to a set of sonographers more adept than others may result in higher efficiency and a decrease in unnecessary further testing for the patient.

The study has multiple limitations which need to be acknowledged. We are a relative small center which resulted in variable and sometimes insufficient number of cases for adequate assessment of each sonographer and an overall small sample volume. The body mass index (BMI) was not utilized, which may affect the ability to see the appendix. As the patients were referred to the department without direction to a specific sonographer it was felt that over time the differences in BMI would likely equalize out among the scanners. Further limitations were a potential bias related to the sonographer choosing not to perform a ‘query’ appendicitis case deferring to a sonographer with more confidence if possible. In addition, the ability of the staff radiologists to perceive the normal appendix was not assessed. Given, we are at an academic center, the majority of the ER cases sent to the department were initially performed by the trained sonographers who then present the case to the radiology resident on service. The cases may not be reviewed initially by the staff unless a concern or question is raised by the resident. Additionally, the cases are often performed after hours with the resident on-call or available sonographer. It is possible that availability of staff radiologists may have improved detection of the normal appendix. Lastly, a further limitation was utilizing the initial sonography image recorded on the PACS images as the start of the exam as this may not take into account the full time the sonographer took to perform a “scout” scan before acquiring images.

## Conclusion

Similar to previous studies involving mammography and chest imaging modalities, perception/diagnostic error in sonographic image acquisition and interpretation should be recognized. The lack of perception of a normal appendix has the potential to adversely affect patient care and resource allocation. Further research endeavors to better understand, train and test for individual differences in perception ability and the complex cognitive processes involved in image interpretation are needed. Triaging of patients and their related imaging to those sonographers who have displayed the optimal perceptual ability for the suspected disease process may help optimize patient care and hospital resources. Sonography of pediatric patients is most successful when performed by experienced sonographers with adequate allotted time for the study with a history of successful perception of the normal appendix. Having experienced sonographers available after hours would allow for optimal care in the setting of ‘query’ appendicitis.

## Data Availability

The datasets used and/or analyzed during the current study are available from the corresponding author on reasonable request.

## Abbreviations

ER: emergency room
PGY: postgraduate year
